# Supplementary material for: Composition of Pinot Noir Wine from Grapevine Red Blotch Disease-Infected Vines Managed with Exogenous Abscisic Acid Applications
Source: Molecules. 2022 Jul 15;27(14):4520. doi: 10.3390/molecules27144520 (PMC9321972; doi:10.3390/molecules27144520)
Supplement: Supplementary file 1 [file molecules-27-04520-s001.zip › molecules-1766893-supplementary.pdf]

Table S1. Compounds annotation of the grapevine red blotch associated wines under ABA vine treatment with high resolution mass spectrometry.

Accepted Description	*m/z*	Retention time (min)	Adducts	Formula	Score	Fragmentation Score	Mass Error (ppm)	Isotope Similarity
Alkaloid								
2-Hydroxyquinoline	146.0592	12.6	M+H	C9H7NO	54.7	86.4	-5.9	94
Norharmane	191.0563	6.3	M+Na	C11H8N2	35.7	0	-9.8	89
Trigonelline	138.0543	9.2	M+H	C7H7NO2	56.2	86.8	-5	99.7
Amino								
Choline	104.1063	5.4	M+H	C5H13NO	55.8	87.7	-6.8	99.1
N-Ethylacetamide	88.075	10.7	M+H, M+Na	C4H9NO	50	61.2	-8.2	97.8
Amino acid								
Glutamic acid	148.0594	4.6	M+H	C5H9NO4	57.3	97.3	-6.7	96.6
L-Histidine	156.0761	4.3	M+H	C6H9N3O2	56.6	95.8	-4.2	92.2
L-Isoleucine	132.1011	6.6	M+H	C6H13NO2	56.9	92.3	-6.2	99.2
L-Proline	116.0699	5.3	M+H	C5H9NO2	58.1	98.1	-6.4	99.7
Pyroglutamic acid	130.049	8	M+H	C5H7NO3	50.2	59.8	-6.4	98.5
Tyrosine	182.0805	6.2	M+H	C9H11NO3	56.8	92.1	-4	96.4
Amino acid derivative								
Carnitine	162.1117	6	M+H	C7H16ClNO3	50.3	85.1	-4.6	71.9
Acetylcarnitine	204.1225	8.7	M+H	C9H17NO4	51.7	64.1	-2.7	97.7
Betaine	118.0854	6.5	M+H, M+Na	C5H11NO2	56.5	91.6	-7	98.7
Deoxycarnitine	146.1169	8.4	M+H	C7H15NO2	55.1	82.5	-4.7	98.6
Hydroxyproline	132.0646	4.7	M+H	C5H9NO3	55.9	97.7	-7	90
Indolelactic acid	206.0805	17.5	M+H-H_2O, M+H	C11H11NO3	55.9	88.4	-3.4	95
L-Carnitine	162.1118	5.3	M+H	C7H15NO3	57.8	95.7	-4.1	98.3
L-Histidinol	142.0968	3.9	M+H-H_2O, M+H	C6H11N3O	56.3	90.7	-5.2	96.6

N-Fructosyl isoleucine	294.1541	7.1	M+H-H$_2$O, M+H, M+Na	C12H23NO7	51.2	64.5	-2	94.1
Saccharopine	277.1391	4.5	M+H-H$_2$O, M+H, M+Na	C11H20N2O6	52.8	69.4	-1	95.8
S-Adenosylmethionine	399.1438	5	M+H	C15H22N6O5S	57.4	97	-1.9	92.5
Serinyl-phenylalanine	253.1177	20.3	M+H	C12H16N2O4	52.4	68.2	-2.2	96.3
Stachydrine	144.1012	9.4	M+H	C7H13NO2	54.4	78.3	-5	99.3
Targinine	189.134	4.6	M+H	C7H16N4O2	55.1	83.1	-3	96.1
Tryptophanol	144.0799	20.1	M+H-H$_2$O, M+H	C10H11NO	54.3	80.9	-5.2	96.4
N,N-Dimethyl-safingol	330.336	24.3	M+H	C20H43NO2	52.9	73.8	-1.9	92.8
Amino alcohol								
2-Diethylaminoethanol	118.1219	30	M+H	C6H15NO	51.7	66.5	-6.1	98.9
Lauryldiethanolamine	274.2734	22.7	M+H	C16H35NO2	57.3	94.9	-2.4	94.7
Carbohydrate								
Cellotetraose	689.2089	4.7	M+H-H$_2$O, M+H, M+NH$_4$, M+Na	C24H42O21	50.5	67.1	-3.2	89
Gentiobiose	365.1048	4.8	M+H-H$_2$O, M+H, M+Na	C12H22O11	55.1	78.5	-1.8	99.3
Isomaltulose	360.149	4.7	M+NH$_4$	C12H22O11	53.6	77.9	-2.9	93.4
Maltotriose	527.1568	4.8	M+H-H$_2$O, M+NH$_4$, M+Na	C18H32O16	53.8	78.5	-2.9	93.7
Fat								
Dihydrosphingosine	302.3047	23.9	M+H-H$_2$O, M+H, M+Na	C18H39NO2	50	53.3	-2.3	99.4
Glycerophosphocholine	258.1096	5.3	M+H, M+Na	C8H20NO6P	55.8	82.1	-1.8	98.8
MG(18:0/0:0/0:0)	359.3144	26	M+H	C21H42O4	54.2	77.8	-3.3	97.2
Phytosphingosine	318.2995	23.6	M+H	C18H39NO3	56.7	87.9	-2.4	98.5
Sphingosine	300.2891	24	M+H, M+Na	C18H37NO2	50.7	58.5	-2.2	97.6

Fatty acid								
5,8,11-Trihydroxyoctadec-9-enoic acid	353.2289	22.9	M+NH$_4$, M+Na	C18H34O5	50.5	62.1	-2.9	93.9
Monopalmitin	353.2652	25.6	M+H-H$_2$O, M+H, M+NH$_4$, M+Na	C19H38O4	51.3	61.6	-3	98.2
Dihydroxyoctadecadienoic acid	313.2364	23.9	M+H	C18H32O4	52.8	74.1	-3.1	93.8
Vaccenic acid	283.2628	26.2	M+H-H$_2$O, M+H	C18H34O2	51.5	71.2	-1.1	87.7
Furan								
Furaneol	129.0536	9.3	M+H-H$_2$O, M+H	C6H8O3	51.3	69.6	-7.9	95.6
2,3-Dihydrobenzofuran	121.064	14.5	M+H-H$_2$O, M+H	C8H8O	56.2	89.3	-6.2	98.9
Furan	69.033	4.8	M+H	C4H4O	50.9	66.4	-7.4	96.2
Heterocyclic								
Lapidin	335.2185	24.5	M+H	C20H30O4	35.3	0	-9.5	86.9
Myristicin	210.1119	13.6	M+H, M+NH$_4$	C11H12O3	38.8	0	-3.1	97.5
Lactone								
4-Deoxytetronic acid	87.0433	12.7	M+H	C4H6O2	56.7	93.8	-8.6	99.4
Methysticin	297.0718	22.9	M+Na	C15H14O5	36.5	0	-5.6	88.9
Organic acid								
Pantothenic acid	220.1174	10.4	M+H-H$_2$O, M+H, M+Na	C9H17NO5	55.1	80	-2.7	98.6
Abscisic acid	265.1427	15.4	M+H-H$_2$O, M+H	C15H20O4	50.7	58.9	-2.6	97.9
Abscisic acid	287.1247	21.8	M+H-H$_2$O, M+H, M+NH$_4$, M+Na	C15H20O4	50.8	60	-2.7	97.1
3-Indoleacrylic acid	188.0699	11.8	M+H, M+NH$_4$	C11H9NO2	50.8	60.1	-3.8	98.3
Cinnamic acid	166.0853	9.5	M+H, M+NH$_4$	C9H8O2	50.4	59.8	-6.2	99.4
Citric acid	215.0157	5.6	M+H-H$_2$O, M+H, M+NH$_4$, M+Na	C6H8O7	54	74.1	-2.5	98.6

Others								
Butanediol	73.0643	7.5	M+H-H_2O, M+H, M+Na	C4H10O2	54.1	78.3	-5.4	98.6
2,5-Dihydroxybenzaldehyde	139.0382	14.5	M+H	C7H6O3	50.7	61.4	-5.9	99
2-Pyrrolidinone	86.0593	4.7	M+H	C4H7NO	51.3	68.4	-8.9	97.8
D-Sorbitol	205.068	4.7	M+H-H_2O, M+H, M+Na	C6H14O6	55.7	84.5	-1.2	95.5
Glutathione	308.0908	5.4	M+H	C10H17N3O6S	50.5	62.6	-0.9	91.1
Muramic acid	234.0969	4.7	M+H-H_2O, M+H	C9H17NO7	51.2	59.7	-1.2	97.7
Syringin	395.13	17.9	M+Na	C17H24O9	52.2	68.7	-3.3	96.4
Trimethylamine	60.0804	29.8	M+H	C3H9N	57.7	99.4	-6.3	96.4
Phenol-anthocyanidin								
Delphinidin	303.0507	19.3	M+H	C15H10O7	51.4	65.8	2.6	94.2
Malvidin	331.0804	18.1	M+H	C17H14O7	56.5	91.9	-2.4	93.5
Malvidin 3-(6''-acetylglucoside)	535.1424	20.3	M+H, M+Na	C25H26O13	50.3	61	-4.2	95.6
Malvidin 3-(6''-p-coumarylglucoside)	639.1685	21.3	M+H, M+Na	C32H30O14	51.7	68.6	-3.6	94.1
Malvidin 3-O-glucoside	493.1323	18.1	M+H	C23H24O12	56.1	90.5	-3.6	94.3
Peonidin 3-(6''-acetylglucoside)	505.132	20	M+H	C24H24O12	52.1	72.6	-4.1	92.6
Peonidin 3-(6''-p-coumarylglucoside)	609.1579	21.1	M+H, M+Na	C31H28O13	51.6	64.2	-4	98.4
Peonidin-3-O-beta-galactoside	463.1219	17.8	M+H	C22H22O11	57.5	93.8	-3.5	97.8
Petunidin 3-(6''-acetylglucoside)	521.127	19.3	M+H	C24H24O13	51.5	68	-3.9	94.2
Phenol-dihydrochalcone								
Phlorizine	459.1234	19.9	M+Na	C21H24O10	37.1	0	-6.4	92.9
Phenol-flavan-3-ols								

Epicatechin	291.0856	16.9	$M+H-H_2O$, M+H, M+Na	$C_{15}H_{14}O_6$	57.9	94.7	-2.5	97.7
Catechin	291.0856	14.5	$M+H-H_2O$, M+H, M+Na	$C_{15}H_{14}O_6$	54.5	76.4	-2.4	98.7
Epigallocatechin	459.0882	18.4	M+H	$C_{22}H_{18}O_{11}$	36.7	0	-8.8	93.4
Procyanidin B1	579.1473	16.6	M+H, M+Na	$C_{30}H_{26}O_{12}$	50.7	65.9	-4.1	92.2
Procyanidin B2	579.1475	14.3	M+H, M+Na	$C_{30}H_{26}O_{12}$	54.8	79.9	-3.8	98.7
Procyanidin dimer B1	579.1477	21.6	M+H	$C_{30}H_{26}O_{12}$	37.6	0	-3.5	92.2
Procyanidin trimer C1	867.2103	15.4	M+H, M+Na	$C_{45}H_{38}O_{18}$	36.9	0	-3.2	88.5
Prodelphinidin dimer B3	611.1391	9.2	M+H	$C_{30}H_{26}O_{14}$	36.7	0	-0.7	84.6
Phenol-flavanones								
Narigenin-7-O-glucoside	457.1089	20.7	$M+H-H_2O$, M+H, M+Na	$C_{21}H_{22}O_{10}$	38.4	0	-3.8	96.5
Phenol-flavones								
Luteolin 4'-O-glucoside	449.1062	15.5	M+H	$C_{21}H_{20}O_{11}$	56.5	93.9	-3.7	93
Phenol-flavonols								
Taxifolin_Isomer	305.0644	18.4	M+H	$C_{15}H_{12}O_7$	55.6	88.3	-3.8	94.1
Dihydrokaempferol	289.0699	20.7	M+H	$C_{15}H_{12}O_6$	54.5	80.9	-2.8	94.9
Dihydromyricetin 3-O-rhamnoside	489.1019	17.5	M+Na	$C_{21}H_{22}O_{12}$	38.5	0	3.4	96.7
Dihydroquercetin_isomer	305.0646	17.5	M+H	$C_{15}H_{12}O_7$	52.5	70.6	-3.2	95.8
Dihydroquercetin 3-O-rhamnoside	473.1037	19.6	$M+H-H_2O$, M+H, M+Na	$C_{21}H_{22}O_{11}$	38.7	0	-3.8	98.1
Hyperoside	487.0833	19.6	M+H, M+Na	$C_{21}H_{20}O_{12}$	58.2	96.7	-3	97.9
Isoquercitrine	465.1012	13.5	M+H, M+Na	$C_{21}H_{20}O_{12}$	51.7	65.6	-3.3	96.8
Isorhamnetin	317.0656	22.7	M+H, M+Na	$C_{16}H_{12}O_7$	51.2	66.5	0.2	89.6
Isorhamnetin-3-O-glucoside	501.0986	20.7	M+H, M+Na	$C_{22}H_{22}O_{12}$	58.3	97.9	-3.7	97.9
Morin	303.0502	20.4	M+H	$C_{15}H_{10}O_7$	49.1	50	1	96.8

Myricetin-3-Galactoside	503.0778	18.5	M+H, M+Na	C21H20O13	57.7	97.4	-3.8	95.6
Quercetin	303.0501	21.4	M+H, M+Na	C15H10O7	56.8	87.5	0.6	97.3
Quercetin-3-Glucuronide	479.0803	19.3	M+H, M+Na	C21H18O13	58.7	98.1	-3.5	99.4
Quercetin-3-Rhamnoside	471.0879	20.4	M+H-H_2O, M+H, M+Na	C21H20O11	56.6	95.6	-4.3	92.5
Rutine	633.1401	19.3	M+H, M+Na	C27H30O16	52	84.2	-4.1	80.5
Taxifolin	305.0645	19.6	M+H-H_2O, M+H	C15H12O7	55.8	87.3	-3.4	95.8
Phenol-other								
Gingerol	295.1889	23.8	M+H, M+Na	C17H26O4	52.8	81.3	-5	88.3
Antiarol	185.0799	19.6	M+H	C9H12O4	50.7	64.5	-5	94.9
Ethyl Syringate	249.0728	23	M+H, M+Na	C11H14O5	53.7	78.6	-2.4	92.6
Syringaresinol	401.1557	21	M+H-H_2O	C22H26O8	36	0	-9	90.2
Syringetine-3-glucoside	509.127	20.9	M+H	C23H24O13	56.5	90.2	-3.9	96.9
Vannilylacetone	195.1007	24.8	M+H, M+Na	C11H14O3	56.4	92.9	-4.5	94.1
Phenol-phenolic acids								
4,5-Dicaffeoylquinic acid	517.1314	19.7	M+H	C25H24O12	39	0	-4.1	95
4-Coumaric acid	147.0433	18.6	M+H-H_2O, M+H	C9H8O3	57.2	95.8	-4.6	95.6
Caffeoyl tartaric acid	335.0366	11.7	M+H, M+Na	C13H12O9	39.1	0	-2.5	98.4
Coumaric acid	147.0433	14.9	M+H-H_2O	C9H8O3	52	66.4	-4.8	99
p-Coumaroyl tartaric acid	279.047	4.5	M+H-H_2O	C13H12O8	36.2	0	-9.9	91.6
Vanillic acid	186.0754	5.1	M+NH_4	C8H8O4	37.5	0	-3.9	91.9
Phenol-stilbenes								
Deoxykhivorin	593.2736	28.8	M+Na	C32H42O9	54.4	77.9	2.6	97.3
e-Viniferin	455.1473	20.2	M+H	C28H22O6	37.3	0	-3.6	90.6
Resveratrol	229.0852	21.4	M+H	C14H12O3	56.4	90.4	-3	95.4

Resveratrol 3-O-glucoside	413.1193	20.4	M+H, M+NH_4, M+Na	C20H22O8	38.8	0	-3.4	97.9
Trans-piceid	413.1194	18.9	M+H, M+Na	C20H22O8	51.2	65.6	-3.3	94.1
Purine								
2`-O-Methylguanosine	298.1139	11.2	M+H, M+Na	C11H15N5O5	54.3	79.5	-2.3	94.8
2-O-Methyladenosine	282.1191	13.2	M+H	C11H15N5O4	58.7	97.5	-2.2	98.8
5'-S-Methylthioadenosine	298.0961	16.1	M+H	C11H15N5O3S	57.3	95.3	-2.5	94.4
7-Methylguanine	166.0716	10.3	M+H	C6H7N5O	56.4	91.6	-4.4	95.6
Adenine	136.0609	7.4	M+H	C5H5N5	57.4	96.2	-6.2	97.9
Succinoadenosine	384.1143	11.3	M+H, M+Na	C14H17N5O8	50.7	57	-1.8	98.7
Terpenoid								
Catalpol	363.1251	7.8	M+H	C15H22O10	35.3	0	-9.5	86.9
Zerumbone	219.1737	22.1	M+H	C15H22O	38.6	0	-2.8	96.4
